# Regularity of Breakfast Consumption and Diet: Insights from National Adult Nutrition Survey

**DOI:** 10.3390/nu10111578

**Published:** 2018-10-26

**Authors:** Irina Uzhova, Deirdre Mullally, José L Peñalvo, Eileen R. Gibney

**Affiliations:** 1 Department of Health and Nutritional Sciences, Institute of Technology Sligo, Sligo, Ireland; uzhova.irina@itsligo.ie; 2 Institute of Food and Health, University College Dublin, Belfield, Dublin 4, Ireland; mullallydeirdre@outlook.com; 3 Friedman School of Nutrition Science and Policy, Tufts University, Boston, MA 02153, USA; jose.penalvo@tufts.edu

**Keywords:** breakfast, dietary patterns, indices of diet quality

## Abstract

Breakfast is considered to be one of the most important meals of the day. Its omission has been reported to be associated with increased disease risk, such as obesity, diabetes, and coronary heart disease, as well as unhealthy lifestyle and lower dietary quality. Using data from the National Adult Nutrition Survey (NANS)—a food consumption survey conducted among 1500 Irish men and women over 18 years of age, residing in the Republic of Ireland at the time the survey was conducted—we aimed to characterize breakfast regularity, identify dietary patterns associated with regular breakfast consumption, and assess the nutritional quality of such dietary patterns, using the nutrient-rich food index score NRF9.3. We determined two breakfast regularity categories and assessed dietary quality, by means of adherence to the principal component analysis derived dietary patterns and the NRF9.3 dietary index. Regular breakfast consumers were identified as those who consumed breakfast 3–4 times out of the 4 days of the collection period; such consumers comprised the majority of the population (94.4%). They had the highest adherence to healthier dietary patterns, namely, the “vegetarian” (odds ratio (OR): 2.59: 95% Confidence Interval (CI): 1.40, 4.77), “fish and vegetables” (OR: 2.88: 95% CI: 1.63, 5.10), and “breakfast cereals” (OR: 4.62: 95% CI: 2.43, 8.79) dietary patterns. Breakfast significantly contributed to the daily micronutrient intake by providing, on average, 24% of dietary fiber, 32% of iron, 30% of calcium, 32% of folate, and 37% of riboflavin. The importance of regular breakfast consumption on those who skip breakfast should be highlighted, in order to improve compliance with nutritional recommendations and adherence to a healthy lifestyle.

## 1. Introduction

Breakfast is often referred to as the most important meal of the day and its consumption has been associated with an overall healthy dietary pattern, as measured by adherence to the Mediterranean-type diet [1] or the Healthy Eating Index (HEI) [2]. Breakfast skipping, conversely, has been reported to be associated with several cardiovascular risk factors, including unfavorable lipid profile, obesity, and diabetes, as well as atherosclerosis and coronary heart disease [3,4]. It has been shown also to be related to lower dietary quality [5], unhealthy lifestyle behaviors—such as smoking [6]—and lack of physical activity [7]. Some studies have evaluated the contribution of breakfast to daily macro- and micronutrient intake, reporting that breakfast skippers have significantly lower intake of dietary fiber, niacin, folate, riboflavin, vitamins C and A, calcium, phosphorus, iron, potassium, and magnesium [8,9]. Breakfast skippers were also less likely to comply with estimated average requirements (EAR) for calcium, vitamin C, and folate [10]. To the best of our knowledge, there are no studies which have investigated the middle point between breakfast consumption and breakfast omission, regarding the regularity of breakfast intake. By addressing this knowledge gap, we aim to characterize the regularity of breakfast consumption within an adult population in Ireland, identify the overall dietary patterns associated with breakfast consumption, and evaluate its nutritional quality, using the nutrient-rich food (NRF) index score NRF9.3 [11]. We hypothesized that regular breakfast consumption, compared to irregular breakfast consumption, would be associated with higher dietary quality and overall healthier dietary patterns.

## 2. Materials and Methods 

### 2.1. Study Population

NANS (National Adult Nutrition Survey), is a cross-sectional food consumption survey, conducted among adult Irish participants. Details of the study design and methodology have been previously described [12]. Data on diet, lifestyle, and attitudes toward food were collected for 1500 men and women over 18 years of age, residing in the Republic of Ireland at the time of the survey. Lactating and pregnant women were excluded from the survey. The study protocol was approved by the Human Ethics Research Committee of University College Dublin and the University College Cork Research Ethics Committee of the Cork Teaching Hospitals (ECM 3(p) 04/11/08), and all participants provided informed written consent.

### 2.2. Assessment of Demographic, Dietary, and Other Lifestyle Data 

The demographic information collected included: Sex (male/female), age (years), marital status (married/widow/single/divorced), and education level (secondary school/professional training/higher education). Dietary data were collected by means of a 4-day food diary, in which participants were requested to record and weigh any food or beverage consumed over 4 days and provide information, regarding the type of food and meal consumed, its preparation method, manufacturer, and the time of ingestion. Weighed Intake Software Program WISP version 3.0 (Tinuviel Software, Llanfechell, Anglesey, UK) was used to analyze nutrient intake data. A total of 2552 food codes (including 233 supplements) was categorized into 19 food groups for data analysis. Lifestyle variables examined included: Smoking status (currently smoke/used to smoke/never smoked), physical activity (recreational physical activity (MET h/w)/home activity (MET h/w)) and time spent watching television (h/w). Analysis was conducted for breakfast consumers only; therefore, those participants who skipped breakfast for all 4 days of the food dairy (*n* = 14) were excluded and the final sample for analysis consisted of 1486 participants. Missing values on weight (*n* = 87) were excluded from the NRF9.3 index computation. 

### 2.3. Definition of Breakfast and Categorization of Regular Versus Irregular Breakfast Consumers

As mentioned, the data on the type of meal consumed (breakfast, snack, light meal, main meal) were self-reported by participants. Therefore, in order to be consistent with definitions previously published in the literature, we verified that our breakfast variable was aligned with the one provided by Timlin et al. [13]: “The first meal of the day that breaks the fast after the longest period of sleep, eaten before or at the start of daily activities”; as well as the one described by O’Neil et al. [14]: “A food or beverage from at least one food group. Coffee, water and non-alcoholic beverages are not included in a food group.” 

To determine the regularity of breakfast consumption, the number of days in which breakfast was consumed, during the 4-day collection period, were classified as follows: 4/4—breakfast consumed on 4 out of 4 days; 3/4—breakfast consumed on 3 out of 4 days; 2/4—breakfast consumed on 2 out of 4 days; and 1/4—breakfast consumed 1 out of 4 days. Regular and irregular breakfast consumers were defined as consuming breakfast 3–4 out of 4 days and 1–2 out of 4 days, respectively. 

### 2.4. Food-based Patterns, Meal Intake Assessment and Calculation of Nutrient-Rich Food Index Score NRF9.3

The overall food-based dietary patterns followed by participants of the study were identified by principal component analysis (PCA). This method was used to identify common underlying dimensions (factors) of food consumption, by deriving factor loadings for each pre-defined food group. Factors were subsequently rotated, using a varimax procedure, to maintain uncorrelated factors. Analysis of eigenvalues, scree plot, and the interpretability of the factor solution were used to support a final decision on retaining a 7-factor solution. A factor score for each participant was calculated by summing the daily intake of each food group and weighting it by their factor loadings. Further, the factor score of each of the 7 dietary patterns was split, according to the tertiles—which were also used in the analysis—whereby the highest tertile indicated highest adherence to a specific dietary pattern. 

To assess how breakfast regularity related to the type of meal consumed during the remainder of the day, we used a generic meal coding system, previously applied to the NANS dataset. The methodology has been described in detail elsewhere [15,16]. The available dietary data on food items was reduced to 20 pre-defined food categories, with meal types corresponding to each of those categories. Among them, light meals (throughout the day) and main meals (including lunch and dinner) were used in the analysis. Within each meal type, the combination of all food groups consumed by one person on a single survey day was identified as an individual meal. The individual meals were grouped into generic categories, including 6 generic light meal categories (skipped light meal, meat/fish/dairy sandwich, dairy sandwich, meat or fish sandwich, soups or salads, rice/potato/past and, other) and 4 generic main meals (skipped main meal, protein- and carbohydrate-based, protein-based, and carbohydrate-based main meals) [16].

To assess the overall nutritional quality of the dietary patterns derived, we used the Nutrient-rich food index NRF9.3 [11], which included 9 nutrients of which intake were encouraged—namely, protein, dietary fiber, vitamins A, C, and E, calcium, iron, potassium, magnesium—and 3 nutrients to limit: Saturated fat, total sugar, and sodium. The recommended daily values (DV) used in the calculation of the index were set by the European Food Safety Authority (EFSA; Panel on Dietetic Products, Nutrition and Allergies), as outlined in the Supplementary Materials, Table S1. The sub-scores for nutrient content in 100 g of a food group (NRn 100 g) and the content of limiting nutrients in 100 g of a food group (LIM 100 g) were calculated for each of the 18 food groups (nutritional supplements were excluded), as shown in the Supplementary Materials, Table S2. The percentage of reference DV for each nutrient was capped at 100% of DV to avoid overvaluing food items. The values of NRn 100 g and LIM 100 g were converted into the variable NRn 100 kcal (content of nutrient in 100 kcal of the selected food group) and into the variable LIM 100 kcal (content of limiting nutrient in 100 kcal of selected food group). The NRF9.3 score for each food group was calculated by subtracting LIM 100 kcal from NRn 100 kcal. Following the methodology applied by Sluik et al. [17], NRF9.3 food scores, per food group, were converted to individual NRF9.3 index scores. The energy consumed from each food group (in 100 kcal units) was multiplied by the NRF9.3 score calculated for each food group. The scores obtained were summed together and divided by the number of 100 kcal units of participants’ total energy intake. The NRF9.3 index was used to determine the nutritional quality of regular breakfast consumption and the dietary patterns derived from PCA.

### 2.5. Statistical Analysis

An ANOVA test for continuous variables and a chi-square test for categorical variables were used to compare the data between the categories of breakfast consumers. Characteristics of the study population were presented as counts, percentages for categorical variables, and mean and SD for continuous variables. Non-adjusted logistic regression models were used to assess the association between adherence to PCA-derived dietary patterns (3rd tertile vs. 1st tertile) and the regularity of breakfast consumption. Additionally, to assess the relationship between overall dietary quality, measured by the nutritional index NRF 9.3, and adherence to dietary patterns identified by PCA, the Pearson correlation coefficient was used. The *p* values < 0.05 (two-sided) found were considered statistically significant. All statistical analyses were performed, using the IBM SPSS Statistics for Windows, version 24 (IBM Corp., Armonk, NY, USA).

## 3. Results

Of the 1486 participants in the study, 94% consumed breakfast on at least 3 days out of the 4-day collection period and were considered regular breakfast consumers, as shown in Table 1.

Participants of all age groups were equally represented, with slightly fewer participants being over 50 and 64 years of age (21% and 15%, respectively). A total of 46% of the cohort reported a higher level of education, 58% reported being married, and 52% reported to never have smoked. Nearly 40% of the participants preferred to skip a light meal and the majority of the population (93%) consumed a main meal, containing protein- and carbohydrate-based food groups. With respect to breakfast regularity, significant differences were observed according to gender, age, marital status, smoking habits, time spent watching television, and the type of light meal consumed during the day, between irregular and regular breakfast consumers. Approximately half (52%) of the regular breakfast consumers were females. Younger adults (18–35 year) were more likely to be irregular breakfast consumers, while the older population (50 years of age and older) were most likely to consume breakfast regularly. No difference was observed in the education level of regular and irregular breakfast consumers. Single participants and those who currently smoke were most likely to be irregular breakfast consumers. Though no difference was observed between regular and irregular breakfast consumers, in terms of the level of recreational and at-home physical activity, those who consumed breakfast regularly spent significantly less time watching television. Irregular breakfast consumers were more likely to skip the consumption of a light meal, while regular breakfast consumers were more likely to consume a sandwich as a light meal. The assessment of overall dietary quality showed that regular breakfast consumers, compared to irregular breakfast consumers, had a significantly higher NRF 9.3 score. 

Overall, it was observed that breakfast provided 19.9% of total daily energy intake, as well as 24.4% of dietary fiber, 31.6% of iron, 29.8% of calcium, 32.0% of folate, and 36.5% of B2 intakes. Compared to regular breakfast consumers, irregular breakfast consumers had significantly (*p* < 0.05) lower intakes of dietary fiber, iron, calcium, folate, B2, and vitamin D, as well as higher intake of sodium and fat at breakfast, as shown in Table 2. Assessment of energy and nutrient intake at breakfast, from the major food groups, showed that breakfast cereals, breads and rolls, dairy, and meat dishes accounted for 22.8%, 19.8%, 11.2%, and 10.0% of energy, respectively. This resulted in a total contribution of 63.8% of energy, 74.5% of protein, 46.2% of fat, 70.5% of carbohydrates, and 71.6% of dietary fiber at breakfast, as shown in the Supplementary Materials, Table S3. Fruits, table sugar, confectionary, preserves, savory snacks, biscuits, cakes, milk, and yogurt contributed to 71.1% of total sugar intake. It was also found that 52.8% of fat intake was derived from butter, spreads, oils, meats, and eggs. Nutritional supplements did not contribute significantly to the intake of energy or macronutrients, accounting for less than 1% of energy, fats, and carbohydrates, and 1.77% of protein. However, they were a major source of micronutrients, providing 66.2% of vitamin B1, 58.7% B2, and 58.8% of B12 intakes, as shown in the Supplementary Materials, Table S4. Exclusion of nutritional supplements from the analysis revealed that breakfast cereals were the main source of micronutrients, including iron, folate, B1, B2, and B3, contributing 56.3%, 39.0%, 50.4%, 44.3%, and 56.0%, respectively, of each micronutrient’s total intake. Dairy was the main source of calcium (41.7%) and vitamin B12 (30.4%), as shown in the Supplementary Materials, Table S5. Assessment of baseline clinical characteristics showed no significant difference between regular and irregular breakfast consumers, except for high-density lipoprotein (HDL-c), which was 0.11 mmol/l lower in irregular breakfast consumers, as shown in the Supplementary Materials, Table S6. 

**Figure 1:**
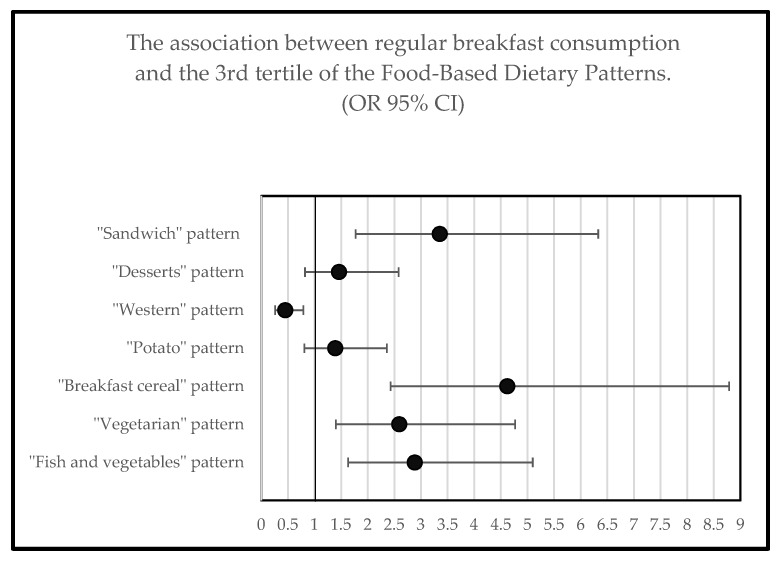
The association between regular breakfast consumption and the highest adherence to the food-based dietary patterns (3rd tertile). Non-adjusted logistic regression model with odds ratios (OR) and 95% confidence interval (CI).

A total of four dietary patterns were most likely to be followed by regular breakfast consumers: the “Sandwich”, “Breakfast cereal”, “Fish and vegetables”, and “Vegetarian” dietary patterns. The “Sandwich” pattern was characterized by higher intakes of breads and rolls, butter, oils, and cheese. According to the NRF9.3, greater adherence to this dietary pattern was associated with the lowest nutritional quality. The “breakfast cereal” pattern was characterized by higher intakes of breakfast cereals, dairy, nuts, and seeds. Higher adherence to the “Vegetarian” dietary pattern was correlated with increased consumption of nuts, seeds, herbs, soups, fruits, vegetables, and nutritional supplements. The “Fish and vegetables” pattern was strongly correlated with higher intakes of fish and vegetables, as well as lower intakes of meat, showing a moderate positive linear relationship with NRF 9.3. The “Western” pattern was characterized by higher intakes of grains, pasta, beverages, meat, and eggs, and had the lowest probability to be followed by regular breakfast consumers (odds ratio (OR): 0.45: 95% CI: 0.26, 0.79; *p* = 0.006), as shown in Table 3 and Figure 1.

## 4. Discussion

Regular breakfast consumption was associated with overall higher dietary quality, in our study. Participants who consumed breakfast regularly had the highest adherence to the healthiest dietary patterns, namely, the “Vegetarian”, “Fish and vegetables,” and “Breakfast cereals” ones. In contrast to irregular breakfast consumption, regular breakfast consumption, as observed in the NANS, contributed significantly to the total micronutrients profile of participents. It provided, on average, as much as 24% of dietary fiber, 32% of iron, 30% of calcium, 32% of folate, and 37% of riboflavin to the daily micronutrient intakes of participents.

In our study, examination of the different types of dietary patterns, followed by participants who consumed breakfast on most of the days of the survey, showed that they adhered to a healthier diet, as indicated by the types of foods consumed. This also included higher intakes of dairy, breakfast cereals, nuts, seeds, fruits and vegetables, fish, and nutritional supplements, as well as lower intakes of red meat and sugar confectionery. These findings are in line with the results of Hopkins et al. [2], who found that a higher frequency of breakfast consumption was associated with higher intakes of beans, green vegetables, whole grains, and fruits, which are known for their beneficial health properties [18,19]. We also observed that frequent breakfast consumers predominantly reported to choose cereal or toast for breakfast, known to be a good source of fiber, iron, and several B vitamins, as well as dairy—a major source of calcium and vitamin B12. These findings are important, as they highlight that regular breakfast consumption could serve as a simple message from health professionals to ensure optimal nutrition profiles in the public and help the population in meeting its overall daily micronutrient targets. Additionally, studies evaluating the relationship between regular breakfast consumption and regulation of appetite [20,21] have reported that breakfast cereals are potentially satiating and may have a beneficial effect on appetite regulation. This could possibly explain their positive impact on the daily meal consumption pattern. As we observed, those who consumed breakfast more frequently were less likely to skip light meals. As we hypothesized, participants may have chosen the latter dietary intakes more carefully and consciously, with their preference aimed toward healthier food choices, resulting in a healthier dietary pattern overall. On the other hand, irregular breakfast consumers were observed to have a poorer diet overall, which involved lower intakes of dietary fiber, iron, calcium, folate, vitamin B2, and vitamin D, and higher intakes of sodium and fat at breakfast. Our study also showed that irregular breakfast consumers were more likely to be current smokers and to spend more time watching television. These findings are in agreement with previous studies, in which irregular breakfast intake was associated with smoking [6] and increased daily energy intake (EI) [22] among adults, and with increased television time [23] and non-compliance with healthy eating recommendations among children [24]. The overall dietary pattern followed by irregular breakfast consumers in our study fell predominantly into the “Western” dietary pattern, which was characterized by higher intakes of grains, pasta, savories, beverages, and meat dishes, which previously has been linked to unhealthy lipid profiles, overweightness and obesity, and metabolic syndromes [25,26,27]. In line with this cluster of unhealthy behaviors, irregular breakfast consumption might serve as a marker for a generally unhealthy diet and lifestyle.

A number of studies have investigated whether regular breakfast consumption is associated with a lower prevalence of obesity. For example, young adults in the US National Health and Nutrition Examination Survey (NHANES), who consumed ready-to-eat cereals for breakfast, were 31% less likely to be overweight or obese and 39% less likely to have abdominal obesity, compared to those who skipped breakfast [9]. Additionally, a meta-analysis of 19 studies showed a 75% increase in the risk of obesity among irregular breakfast consumers [28]. In a recent study by Megson et al., the authors reported that increased frequency of breakfast consumption was associated with significantly higher weight loss, in a sample of adults enrolled in an obesity treatment program [29]. In our study, irregular breakfast consumers were observed to have significantly higher weights, compared to regular breakfast consumers. However, due to the nature of our analysis, we could not rule out the possibility that overweight individuals might have engaged in irregular breakfast consumption, in order to lose weight.

Our study has some limitations and strengths worth considering. The participants of the NANS were predominantly white Irish, which might imply specific dietary patterns. As such, the results presented here might not be generalizable to other populations. Further research in a more heterogeneous population is needed to confirm our findings. The analyses applied in our study were cross-sectional in nature; therefore, it could be of interest for future studies to investigate whether the dietary patterns followed by irregular and regular breakfast consumers are constant over time. It is also worth considering that, currently, there is no universal definition of “breakfast consumption.” Therefore, suggestions for future direction should include the validation of our definiton of breakfast regularity and investigation into the relationship between regular and irregular breakfast intake and chronic disease progression. Our study has several strengths. The cohort included a large, nationally-representative sample of Irish men and women, as well as a thorough examination of diet, which were both unique features of the NANS. Very few studies have collected data capturing dietary intake, during specific eating occasions. This feature allowed us to categorize the regularity of breakfast intakes and to study the relationship, not only of food-based dietary patterns, but, more importantly, of the meals consumed throughout the day. 

In conclusion, our study suggests that regular breakfast intake is associated with overall higher dietary quality, lower prevalence of smoking, and decreased television watching time. Therefore, our study could serve as an indicator of healthy dietary and lifestyle behavior. Based on the findings obtained, it could be suggested that health professionals highlight the importance of regular breakfast consumption to those who skip breakfast. This simple message could improve compliance with nutritional recommendations and adherence to a healthy lifestyle.

## Figures and Tables

**Table 1:** Demographic and lifestyle characteristics of regular and irregular breakfast consumers, from a 4-day national dietary survey of Irish adults (*n* = 1486).

Baseline Characteristics	Total *n* = 1486	Irregular *n* = 83	Regular *n* = 1403
**Sex**			
Male	731 (49.2)	51 (61.4)	680 (48.5) *
Female	755 (50.8)	32 (38.6)	723 (51.5) *
**Age Group (Years)**			
18–35	526 (35.4)	58 (69.9)	468 (33.4) *
36–50	431 (29.0)	18 (21.7)	413 (29.4) *
50–64	304 (20.5)	7 (8.40)	297 (21.2) *
>64	225 (15.1)	0 (0.00)	225 (16.0) *
**Education**			
Secondary school	480 (33.4)	32 (42.7)	448 (32.9)
Professional training	293 (20.4)	10 (13.3)	283 (20.8)
Higher education	663 (46.2)	33 (44.0)	630 (46.3)
**Marital Status**			
Single	496 (32.3)	47 (62.7)	422 (30.6) *
Married	849 (58.4)	27 (36.0)	822 (59.6) *
Widowed	71 (4.90)	0 (0.00)	71 (5.10) *
Separated	65 (4.50)	1 (1.30)	64 (4.60)
**Smoking Habits**			
Current smoker	290 (20.1)	28 (36.8)	262 (19.1) *
Former smoker	397 (27.5)	14 (18.4)	383 (28.0) *
Never smoked	759 (52.5)	34 (44.7)	725 (52.9) *
**Physical Activity**			
Recreational physical activity (MET h/w)	35.8 ± 67.9	39.8 ± 44.2	35.5 ± 69.1
Home activity (MET h/w)	43.9 ± 38.3	42.3 ± 41.7	44.0 ± 38.1
Watching television (h/w)	19.7 ± 10.2	22.4 ± 9.90	19.5 ± 10.2 *
**Anthropometry**			
BMI (kg/m^2^)	27.1 ± 4.98	27.4 ± 5.84	27.1 ± 4.92
Weight (kg)	77.5 ± 16.1	81.2 ± 19.3	77.3 ± 15.9 *
Waist-to-hip ratio	0.88 ± 0.09	0.88 ± 0.09	0.88 ± 0.09
**Light Meal Types**			
Skipped light meal	199 (39.3)	23 (79.3)	176 (36.9) *
MFD sandwich	45 (8.90)	3 (10.3)	42 (8.80)
Dairy sandwich	17 (3.40)	0 (0.00)	17 (3.60)
MF sandwich	140 (27.7)	3 (10.3)	137 (28.7) *
Soup and salad	13 (2.60)	0 (0.00)	13 (2.70)
Rice/potato/pasta	1 (0.20)	0 (0.00)	1 (0.20)
**Main Meal Types**			
Skipped main meal	10 (1.00)	1 (2.00)	9 (0.90)
Protein and carbohydrates	943 (92.9)	44 (86.3)	899 (93.3)
Protein	43 (4.20)	5 (9.80)	38 (3.90)
Carbohydrate	16 (1.60)	1 (2.00)	15 (1.60)
**Dietary Quality**			
NRF 9.3	33.8 ± 14.2	28.5 ± 11.1	34.1 ± 14.3 *

Values presented as n (%), or mean ± SD. Regular breakfast consumers were defined as consuming breakfast on 3–4 days out of 4 and irregular breakfast consumers defined as consuming breakfast 1–2 days out of 4. * signifies *p* < 0.05 for irregular breakfast consumers. Type of meal category allocated to participants was based on their consuming this type of meal on at least 3 out of the 4 days. BMI = body mass index; MF = meat or fish; MFD = meat/fish and dairy.

**Table 2:** Mean daily intakes of energy, macronutrients and micronutrients, at breakfast and overall, among breakfast consumers (*n* = 1486) from a national dietary survey of Irish adults.

Nutrient	Total Breakfast Consumers *n* = 1486			Regular Breakfast Consumers *n* = 1403			Irregular Breakfast Consumers *n* = 83		
	BF Intake	Daily Intake	BF % Daily	BF Intake	Daily Intake	BF % Daily	BF Intake	Daily Intake	BF % Daily
	Mean ± SD or %								
Energy, kcal	400 ± 193	2011 ± 657	19.9	399 ± 186	2004 ± 646	19.9	414 ± 288	2130 ± 800	19.4
Protein, g	13.7 ± 7.35	83.4 ± 26.9	16.4	13.7 ± 7.10	83.6 ± 26.7	16.4	14.4 ± 11.2	80.8 ± 31.4	17.8
Fat, g	12.8 ± 9.70	75.8 ± 29.4	16.9	12.6 ± 9.20	75.6 ± 29.2	16.7	15.6 ±15.9 *	79.7 ± 32.4	19.6
Saturated fat, g	5.15 ± 4.13	29.8 ± 12.9	17.3	5.29 ± 4.16	29.8 ± 12.9	17.8	2.77 ± 2.76 *	30.1 ± 12.8	9.20
Carbohydrates, g	59.0 ± 28.5	229 ± 78.9	25.8	59.2 ± 28.1	229 ± 78.7	25.9	54.7 ± 34.6	219 ± 81.9	25.0
Total sugars, g	24.6 ± 16.1	90.5 ± 43.1	27.2	24.9 ± 15.8	90.8 ± 42.9	27.4	20.3 ± 20.4 *	85.1 ± 46.6	23.9
Dietary fiber, g	4.68 ± 3.55	19.2 ± 7.87	24.4	4.77 ± 3.57	19.5 ± 7.88	24.5	3.21 ± 2.73 *	14.8 ± 6.32 *	21.7
Iron, mg	3.82 ± 3.29	12.1 ± 5.14	31.6	3.82 ± 3.22	12.2 ± 5.12	31.3	3.74 ± 4.33	10.9 ± 5.27 *	34.3
Calcium, mg	268 ± 151	900 ± 371	29.8	268 ± 149	905 ± 370	29.7	252 ± 182	813 ± 379 *	31.0
Sodium, mg	469 ± 366	2501 ± 903	18.8	457 ± 328	2494 ± 898	18.4	650 ± 740 *	2611 ± 982	24.9
Folate, µg	102 ± 81.0	319 ± 153	32.0	102 ± 79.8	322 ± 152	31.7	95.3 ± 99.7	273 ± 145 *	34.9
Vitamin B1, mg	0.48 ± 0.35	1.76 ± 2.21	27.3	0.48 ± 0.34	1.78 ± 2.26	27.0	0.47 ± 0.49	1.42 ± 0.69	33.1
Vitamin B2, mg	0.69 ± 0.50	1.89 ± 0.83	36.5	0.69 ± 0.49	1.91 ± 0.84	36.1	0.60 ± 0.69	1.60 ± 0.72 *	37.5
Vitamin B3, mg	5.02 ± 4.45	24.2 ± 10.7	20.7	5.02 ± 4.37	24.2 ± 10.6	20.7	5.08 ± 5.59	24.5 ± 11.8	20.7
Vitamin B12, µg	0.97 ± 0.87	4.65 ± 3.5	20.9	0.97 ± 0.86	4.69 ± 3.53	20.7	0.92 ± 0.90	4.04 ± 2.88	22.8
Vitamin D, µg	0.81 ± 1.15	3.26 ± 2.62	24.8	0.80 ± 1.15	3.30 ± 2.63	24.2	0.90 ± 1.19	2.52 ± 2.30 *	35.7

Regular breakfast consumers defined as consuming breakfast 3–4 days out of 4 and irregular breakfast consumers defined as consuming breakfast 1–2 days out of 4. * signifies value was significantly different from regular breakfast consumers (*p* < 0.05). “BF % daily” signifies contribution of breakfast toward daily energy and nutrient intake. BF = breakfast.

**Table 3:** A representation* of the main food items that characterized each dietary pattern and its correlation with nutritional index NRF 9.3.

Food Groups	“Sandwich”	“Dessert”	“Western”	“Potato”	“Breakfast Cereal”	“Vegetarian”	“Fish and Vegetables”
	Factor Loading						
Bread and rolls	0.819						
Butter and oils	0.795						
Cheese	0.391			−0.512			
Potatoes				0.748			
Baked goods		0.708					
Sugar/confectionary/snacks		0.639					−0.305
Desserts		0.513					
Beverages			0.708				
Meat			0.526				−0.465
Grains, pasta, and savories			0.485	−0.440			
Eggs			0.315		−0.370		
Dairy					0.668		
Breakfast cereal					0.534		
Nuts, seeds, and herbs					0.366	0.529	
Supplements						0.654	
Soups and sauces						0.532	
Fruits						0.522	
Vegetables						0.320	0.568
Fish							0.740
	Correlation Coefficient Between Food–Based Dietary Patterns and NRF 9.3						
NRF 9.3	−0.332 **	−0.282 **	−0.165 **	−0.036	0.072 **	0.283 **	0.357 **

* Values correspond to factor loadings (the higher the value, the more a food item contributed to the dietary cluster). Factor loadings below or above 0.03 were omitted for better interpretability. ** signifies *p* < 0.01.

## Supplementary Materials

The following are available online at http://www.mdpi.com/2072-6643/10/11/1578/s1, Table S1: Recommended daily values (RDV) and maximum daily values (MDV), based on a 2000 kcal per day intake, for selected nutrients by the European Food Safety Authority (EFSA); Table S2: Overview of the nutrient rich foods NRF 9.3 score algorithm; Table S3: Intakes of energy and macronutrients (units/day) from breakfast and their contribution (%) to the total intakes at breakfast from major food groups, for breakfast consumers from a national dietary survey of Irish adults *(n* = 1486); Table S4: Intake of micronutrients (units/day) from breakfast and their contribution (%) to the total intakes at breakfast from major food groups, among breakfast consumers (*n* = 1486) from a national dietary survey of Irish adults; Table S5: Intake of micronutrients (units/day) from breakfast and their contribution (%) to the total intakes at breakfast from major food groups, excluding nutritional supplements, among breakfast consumers (*n* = 1486) from a national dietary survey of Irish adults; Table S6: Baseline clinical characteristics of regular and irregular breakfast consumers from a 4-day national dietary survey of Irish adults (*n* = 1486).
