# Supplementary material for: Regularity of Breakfast Consumption and Diet: Insights from National Adult Nutrition Survey
Source: Nutrients. 2018 Oct 26;10(11):1578. doi: 10.3390/nu10111578 (PMC6267347; doi:10.3390/nu10111578)
Supplement: Supplementary file 1 [file nutrients-10-01578-s001.pdf]

Supplementary Materials

Table S1. Recommended daily values (RDV) and maximum daily values (MDV), based on a 2000 kcal per day intake, for selected nutrients by the European Food Safety Authority (EFSA).

Nutrients	DV	MDV
**Nutrients to Encourage**		
Protein (g)	0.83 per kg of body mass *	
Fiber (g)	25 *	
Vitamin A (RE)	750 – men *<br>650 – women *	
Vitamin C (mg)	111 – men *<br>95 – women *	
Calcium (mg)	950 *<br>1000 – 18–24 years *	
Iron (mg)	11 *<br>16/11 – pre-/postmenopausal *	
Vitamin E (mg)	13 – men *<br>11 – women *	
Potassium (mg)	3500 *	
Magnesium (mg)	350 – men *<br>300 – women *	
Nutrients to limit		
Saturated fat (g)		20 *
Total sugar (g)		125 *
Sodium (mg)		2400 *

* EFSA Panel on Dietetic Products, Nutrition and Allergies.

Table S2. Overview of the nutrient rich foods NRF 9.3 score algorithm.

Model	Algorithm	Comment
NR100 g	$\sum_{i=1}^{9}(Nutrient_i / DV_i) \times 100$	$Nutrient_i$ = content of nutrient in 100 g of selected food group.
NR100 kcal	(NR100 g / ED) × 100	
LIM3100 g	$\sum_{i=1}^{9}(L_i / MDV_i) \times 100$	L_i = content of limiting nutrient in 100 g of selected food group.
LIM3100 kcal	(LIM3100 g / ED) × 100	
NRF9.3100 kcal	NR100 kcal–LIM3100 kcal	

DV = recommended daily value; MDV = maximum daily value; ED = energy density (kcal/100 g).

Table S3. Intakes of energy and macronutrients (units/day) from breakfast and their contribution (%) to the total intakes at breakfast from major food groups, for breakfast consumers from a national dietary survey of Irish adults (n = 1486).

Food Group	Energy	Protein	Fat	Saturated fat	Carbohydrate	Total sugar	Fiber
	kcal/day or g/day (% of contribution to total intake)						
Actual mean daily population intake	402	14.0	12.9	5.15	59.1	24.6	4.73
Sum of the food groups	608 (100)	21.5 (100)	21.6 (100)	9.00 (100)	83.1 (100)	33.9 (100)	6.69 (100)
Breakfast cereals	138.6 (22.8)	4.24 (19.7)	2.09 (9.76)	0.69 (7.67)	27.2 (32.7)	5.70 (16.8)	2.41 (36.0)
Bread and rolls	120.2 (19.8)	4.35 (20.3)	1.43 (6.61)	0.50 (5.56)	23.0 (27.7)	1.92 (5.66)	2.22 (33.2)
Milk and yoghurt	68.3 (11.2)	4.11 (19.1)	2.84 (13.1)	1.75 (19.4)	6.51 (7.83)	6.39 (18.8)	0.03 (0.45)
Butter, spreads, and oils	48.8 (8.02)	0.07 (0.33)	5.37 (24.8)	2.56 (28.4)	0.10 (0.12)	0.06 (0.18)	0.00 (0.00)
Fruit and fruit dishes	43.2 (7.10)	0.57 (2.65)	0.13 (0.60)	0.02 (0.22)	10.5 (12.6)	10.1 (29.8)	1.08 (16.1)
Sugars, confectionary, preserves, and savory snacks	30.4 (5.00)	0.11 (0.51)	0.33 (1.53)	0.13 (1.44)	7.20 (8.66)	6.64 (19.6)	0.08 (1.20)
Meat and meat products	60.6 (10.0)	3.30 (15.4)	3.60 (16.7)	1.34 (14.9)	1.91 (2.30)	0.18 (0.53)	0.13 (1.94)
Eggs and egg dishes	31.2 (5.13)	2.25 (10.5)	2.45 (11.3)	0.80 (8.89)	0.02 (0.02)	0.02 (0.06)	0.00 (0.00)
Grains, rice, pasta, and savories	11.7 (1.92)	0.37 (1.72)	0.49 (2.27)	0.13 (1.44)	1.49 (1.79)	0.39 (1.15)	0.13 (1.94)
Biscuits, cakes, and pastries	16.0 (2.63)	0.27 (1.26)	0.76 (3.52)	0.32 (3.56)	2.33 (2.80)	0.97 (2.86)	0.13 (1.94)
Cheeses	8.69 (1.43)	0.53 (2.47)	0.69 (3.19)	0.43 (4.78)	0.03 (0.04)	0.03 (0.09)	0.00 (0.00)
Beverages	3.32 (0.55)	0.19 (0.88)	0.01 (0.05)	0.01 (0.11)	0.73 (0.88)	0.65 (1.92)	0.00 (0.00)
Supplements	3.52 (0.58)	0.38 (1.77)	0.16 (0.74)	0.02 (0.22)	0.11 (0.13)	0.06 (0.18)	0.05 (0.75)
Other food groups *	23.8 (3.91)	0.74 (3.45)	1.27 (5.87)	0.30 (3.31)	1.98 (2.38)	0.83 (2.45)	0.43 (6.43)

* Other food groups (n = 6) included “creams, ice-creams, and desserts”, “potato and potato dishes”, “vegetable and vegetable dishes”, “fish and fish dishes”, “soups, sauces, and miscellaneous foods”, and “nuts, seeds, herbs, and spices”.

Table S4. Intake of micronutrients (units/day) from breakfast and their contribution (%) to the total intakes at breakfast from major food groups, among breakfast consumers (n = 1486) from a national dietary survey of Irish adults.

Food Group	Iron	Calcium	Folate	Thiamine	Riboflavin	Niacin	Vitamin B12	Vitamin D
	kcal/day or µg/day (% of contribution to total intake)							
Actual mean daily population intake	6.04	303	147	1.62	1.78	8.29	2.86	1.89
Sum of the food groups	8.11 (100)	397 (100)	202.1 (100)	2.05 (100)	2.21 (100)	11.2 (100)	3.58 (100)	2.52 (100)
Breakfast cereals	3.14 (38.7)	60.4 (15.1)	54.2 (26.8)	0.35 (17.0)	0.41 (18.3)	4.06 (36.3)	0.33 (9.20)	0.32 (12.6)
Bread and rolls	0.99 (12.2)	76.9 (19.3)	21.1 (10.5)	0.13 (6.10)	0.04(1.83)	1.21 (10.8)	0.00 (0.13)	0.02 (0.75)
Milk and yoghurt	0.05 (0.58)	149 (37.6)	19.7 (9.73)	0.04 (2.02)	0.27(12.3)	0.18 (1.61)	0.45 (12.7)	0.22 (8.77)
Butter, spreads, and oils	0.00 (0.00)	0.44 (0.11)	13.7 (6.75)	0.00 (0.07)	0.00(0.15)	0.02 (0.18)	0.10 (2.70)	0.21 (8.44)
Fruit and fruit dishes	0.21 (2.64)	10.7 (2.70)	12.3 (6.07)	0.05 (2.61)	0.03(1.29)	0.35 (3.10)	0.00 (0.12)	0.00 (0.04)
Sugars, confectionary, preserves, and savory snacks	0.07 (0.81)	3.92 (0.98)	0.92 (0.45)	0.01 (0.28)	0.01(0.33)	0.07 (0.65)	0.00 (0.12)	0.00 (0.01)
Meat and meat products	0.39 (4.79)	13.1 (3.28)	1.03 (0.51)	0.06 (3.09)	0.03(1.19)	0.82 (7.34)	0.19 (5.22)	0.14 (5.63)
Eggs and egg dishes	0.34 (4.22)	10.8 (2.72)	6.53 (3.23)	0.01 (0.63)	0.07(2.95)	0.02 (0.15)	0.27 (7.46)	0.31 (12.3)
Grains, rice, pasta, and savories	0.12 (1.45)	2.80 (0.70)	0.78 (0.38)	0.01 (0.60)	0.00(0.19)	0.06 (0.52)	0.01 (0.17)	0.00 (0.18)
Biscuits, cakes, and pastries	0.07 (0.85)	3.59 (0.90)	0.76 (0.38)	0.01 (0.30)	0.00(0.19)	0.04 (0.36)	0.01 (0.18)	0.01 (0.59)
Cheeses	0.01 (0.12)	16.3 (4.10)	0.61 (0.30)	0.00 (0.05)	0.01(0.45)	0.00 (0.02)	0.03 (0.73)	0.01 (0.22)
Beverages	0.02 (0.23)	1.37 (0.34)	4.68 (2.32)	0.00 (0.02)	0.03(1.46)	0.22 (1.96)	0.02 (0.56)	0.00 (0.00)

Supplements	2.54 (31.4)	40.4 (10.1)	62.8 (31.1)	1.36 (66.2)	1.30(58.7)	3.95 (35.3)	2.10 (58.8)	1.25 (49.1)
Other food groups *	0.16 (1.98)	7.27 (1.83)	3.00 (1.49)	0.02 (0.98)	0.01(0.66)	0.20 (1.78)	0.07 (1.97)	0.03 (1.33)

* Other food groups (n = 6) included "creams, ice-creams, and desserts", "potato and potato dishes", "vegetable and vegetable dishes", "fish and fish dishes", "soups, sauces, and miscellaneous foods", and "nuts, seeds, herbs, and spices".

Table S5. Intake of micronutrients (units/day) from breakfast and their contribution (%) to the total intakes at breakfast from major food groups, excluding nutritional supplements, among breakfast consumers (n = 1486) from a national dietary survey of Irish adults.

Food Group	Iron	Calcium	Folate	Thiamine	Riboflavin	Niacin	Vitamin B12	Vitamin D
	kcal/day or µg/day (% of contribution to total intake)							
Actual mean daily population intake	3.82	268	102	0.48	0.69	5.02	0.97	0.81
Sum of the food groups	5.57 (100)	356 (100)	139 (100)	0.69 (100)	0.91 (100)	7.25 (100)	1.48 (100)	1.27 (100)
Breakfast cereals	3.14 (56.3)	60.4 (16.9)	54.2 (39.0)	0.35 (50.4)	0.41(44.3)	4.06 (56.0)	0.33 (22.3)	0.32 (25.2)
Bread and rolls	0.99 (17.7)	76.9 (21.5)	21.1 (15.2)	0.13 (18.0)	0.04(4.42)	1.21 (16.7)	0.00 (0.00)	0.02 (1.57)
Milk and yoghurt	0.05 (0.89)	149 (41.7)	19.6 (14.1)	0.04 (5.97)	0.27(29.8)	0.18 (2.48)	0.45 (30.4)	0.22 (17.3)
Butter, spreads, and oils	0.00 (0.00)	0.44 (0.12)	13.6 (9.80)	0.00 (0.22)	0.00(0.36)	0.02 (0.28)	0.10 (6.76)	0.21(16.5)
Fruit and fruit dishes	0.21 (3.77)	10.7 (3.00)	12.2 (8.81)	0.05 (7.72)	0.03(3.12)	0.35 (4.83)	0.00 (0.00)	0.00 (0.00)
Sugars, confectionary, preserves and savory snacks	0.07 (1.25)	3.92 (1.10)	0.92 (0.66)	0.01 (0.84)	0.01(0.80)	0.07 (0.97)	0.00 (0.00)	0.00 (0.00)
Meat and meat products	0.39 (7.00)	13.1 (3.67)	1.03 (0.74)	0.06 (9.14)	0.03(2.88)	0.82 (11.3)	0.19 (12.8)	0.14 (11.0)
Eggs and egg dishes	0.34 (6.10)	10.8 (3.03)	6.53 (4.69)	0.01 (1.87)	0.07(7.14)	0.02 (0.28)	0.27 (18.2)	0.31 (24.4)
Grains, rice, pasta, and savories	0.12 (2.15)	2.80 (0.79)	0.78 (0.56)	0.01 (1.79)	0.00(0.46)	0.06 (0.83)	0.01 (0.68)	0.00 (0.00)

Biscuits, cakes, and pastries	0.07 (1.25)	3.59 (1.01)	0.76 (0.55)	0.01 (0.89)	0.00(0.47)	0.04 (0.55)	0.01 (0.68)	0.01 (0.79)
Cheeses	0.01 (0.17)	16.3 (4.57)	0.61 (0.44)	0.00 (0.14)	0.01(1.08)	0.00 (0.00)	0.03 (2.03)	0.01 (0.79)
Beverages	0.02 (0.35)	1.37 (0.38)	4.68 (3.36)	0.00 (0.07)	0.03(3.53)	0.22 (3.03)	0.02 (1.35)	0.00 (0.00)
Other food groups *	0.16 (2.87)	7.27 (2.04)	3.00 (2.16)	0.02 (2.90)	0.01(1.60)	0.20 (2.76)	0.07 (4.73)	0.03 (2.36)

* Other food groups (n = 6) included "creams, ice-creams, and desserts", "potato and potato dishes", "vegetable and vegetable dishes", "fish and fish dishes", "soups, sauces, and miscellaneous foods", and "nuts, seeds, herbs, and spices".

Table S6. Baseline clinical characteristics of regular and irregular breakfast consumers from a 4-day national dietary survey of Irish adults (n = 1486).

**Baseline characteristics**	**Total**	**Irregular** ***n*** **= 83**	**Regular** ***n*** **= 1403**
Serum Triglyceride (mmol/l)	1.31 ± 0.77	1.44 ± 0.96	1.3 ± 0.76
Serum Total Cholesterol (mmol/l)	4.93 ± 1.00	4.95 ± 0.88	4.93 ± 1.00
HDL (mmol/l)	1.56 ± 0.43	1.46 ± 0.40	1.57 ± 0.43 *
LDL (mmol/l)	2.78 ± 0.86	2.85 ± 0.79	2.78 ± 0.87
Serum Albumin (g/l)	43.9 ± 2.64	44.1 ± 2.06	43.9 ± 2.67
Serum Glucose (mmol/l)	5.33 ± 1.16	5.07 ± 0.56	5.35 ± 1.19
Serum CRP (mg/l)	2.72 ± 2.91	2.81 ± 2.70	2.71 ± 2.92
Serum Ferritin (mg/ml)	118 ± 111	118 ± 115	118 ± 111
Serum Creatinine (µmol/l)	89.7 ± 14.2	88.7 ± 14.7	89.8 ± 14.2
Hemoglobin (g/dl)	14.2 ± 1.42	14.4 ± 1.45	14.2 ± 1.42
Systolic blood pressure (mmHg)	124 ± 17.81	122 ± 14.9	125 ± 17.9
Diastolic blood pressure (mmHg)	78.0 ± 10.6	76.6 ± 9.37	78.1 ± 10.7

HDL = high density lipoprotein cholesterol; LDL = low density lipoprotein cholesterol; CRP = C-reactive protein.
